# Genomic Analysis of Novel *Sulfitobacter* Bacterial Strains Isolated from Marine Biofilms

**DOI:** 10.3390/md22070289

**Published:** 2024-06-22

**Authors:** Han Cui, Shen Fan, Wei Ding, Weipeng Zhang

**Affiliations:** 1 MOE Key Laboratory of Marine Genetics & Breeding and College of Marine Life Sciences, Ocean University of China, Qingdao 266003, China; cuihan@stu.ouc.edu.cn (H.C.); fs_ouc@stu.ouc.edu.cn (S.F.); 2 MOE Key Laboratory of Evolution & Marine Biodiversity and Institute of Evolution & Marine Biodiversity, Ocean University of China, Qingdao 266003, China; dingwei@ouc.edu.cn

**Keywords:** marine biofilm, *Sulfitobacter*, central metabolism, biosynthetic gene cluster

## Abstract

Bacteria from the genus *Sulfitobacter* are distributed across various marine habitats and play a significant role in sulfur cycling. However, the metabolic features of *Sulfitobacter* inhabiting marine biofilms are still not well understood. Here, complete genomes and paired metatranscriptomes of eight *Sulfitobacter* strains, isolated from biofilms on subtidal stones, have been analyzed to explore their central energy metabolism and potential of secondary metabolite biosynthesis. Based on average nucleotide identity and phylogenetic analysis, the eight strains were classified into six novel species and two novel strains. The reconstruction of the metabolic pathways indicated that all strains had a complete Entner–Doudoroff pathway, pentose phosphate pathway, and diverse pathways for amino acid metabolism, suggesting the presence of an optimized central carbon metabolism. Pangenome analysis further revealed the differences between the gene cluster distribution patterns among the eight strains, suggesting significant functional variation. Moreover, a total of 47 biosynthetic gene clusters were discovered, which were further classified into 37 gene cluster families that showed low similarity with previously documented clusters. Furthermore, metatranscriptomic analysis revealed the expressions of key functional genes involved in the biosynthesis of ribosomal peptides in in situ marine biofilms. Overall, this study sheds new light on the metabolic features, adaptive strategies, and value of genome mining in this group of biofilm-associated *Sulfitobacter* bacteria.

## 1. Introduction

*Sulfitobacter* is a genus of the Roseobacteraceae family, previously known as the *Roseobacter* clade. Many bacterial species of this genus have been extensively studied and have been demonstrated to play key roles in marine biogeochemical cycles. *Sulfitobacter* bacteria were first discovered in 1995 from the H_2_S–O_2_ interface of the Black Sea and were described as Gram-negative, strictly aerobic, heterotrophic bacteria that were capable of sulfite oxidation [1]. Subsequently, *Sulfitobacter* bacteria were isolated from various habitats, including marine sediments, tidal flats, the Arctic, Antarctic Ekho Lake, the Mediterranean Sea, deep seawater, marine phycosphere, coral, and sea grass [2,3,4,5,6]. This suggests that the bacteria of this genus can adapt to a very broad range of marine environments. These bacteria have diverse metabolic capabilities, such as dimethylsulfoniopropionate lysis [2], denitrification [4], photosynthesis [5], and exopolysaccharide production [7]. Accordingly, *Sulfitobacter* strains are used to promote microalgae growth [7] and to protect algae from pathogenic bacteria [8]. While many studies have documented the metabolic features of this genus, *Sulfitobacter* bacteria living in certain marine habitats, such as biofilms on abiotic surfaces, remain unexplored.

In marine environments, biofilms can grow on nearly all substrates immersed in seawater, such as artificial panels [9], animal guts [10], and microplastics [11]. A growing number of studies have recognized the important ecological roles (e.g., mediating interactions between microbes and invertebrates) of marine biofilms [12] and the underestimated biomass and species diversity of these microbiota [13]. A global survey of marine biofilms discovered more than 7300 species and 11 million protein-coding genes that were previously hard to detect in seawater [14]. While these studies have pointed out the importance and mystery of marine biofilms, there is still a lack of studies exploring the metabolic abilities of biofilm-associated microbes. In a recent study [15], 54 novel *Roseobacter* strains were isolated from biofilms on coastal stone surfaces, including eight *Sulfitobacter* strains. The study [15] further explored the metabolic features of isolated strains with a focus on thiosulfate oxidation. These bacterial strains could oxidize thiosulfate to sulfate under both aerobic and anaerobic conditions, demonstrating their unique metabolic features [15]. However, a more comprehensive analysis is required to unveil the genomic features and biosynthetic potential of these bacterial strains.

In the present study, based on the complete genomes of eight *Sulfitobacter* strains isolated from subtidal biofilms, variations among genome content were analyzed along with the reconstruction of metabolic pathways to discover novel biosynthetic gene clusters (BGCs). Furthermore, a metatranscriptomic analysis of marine biofilms was performed to demonstrate the in situ expression of genes encoding key enzymes for the biosynthesis of ribosomal peptides in isolated *Sulfitobacter* strains.

## 2. Results

### 2.1. Phenotype and Phylogeny

**Figure 1:**
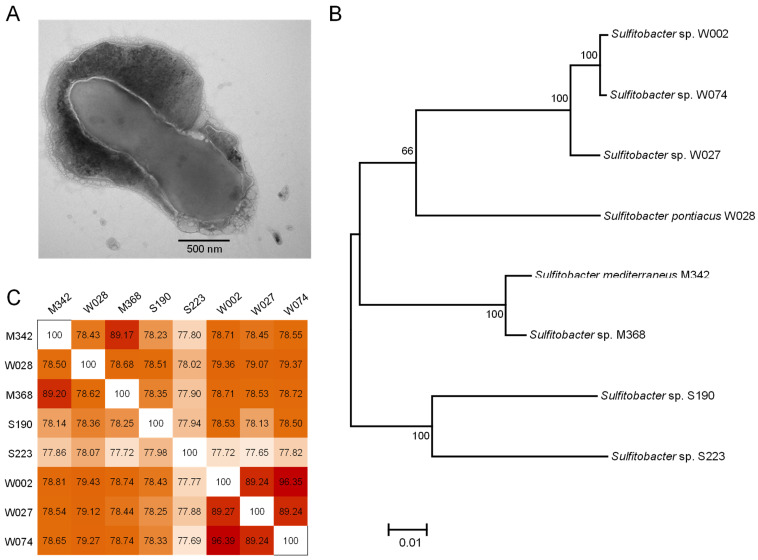
Phenotypes and phylogeny of eight *Sulfitobacter* strains isolated from marine biofilms. (**A**) Footprint-shaped bacterial cells and extracellular matrix observed under a transmission electron microscope. (**B**) Evolutionary relationships of strains revealed by a maximum likelihood tree. (**C**) Pairwise comparison of average nucleotide identities.

All the isolated *Sulfitobacter* strains (strain names: M342, M368, S190, S223, W002, W027, W028, and W074) could grow well, generating a substantial biomass in marine broth 2216E medium. As a result, enough cells could be obtained for the downstream observation, genome sequencing, and analysis of *Sulfitobacter* strains. Under a transmission electron microscope (TEM), the bacterial cells were observed as footprint-shaped cells, with a substantial abundance of matrix around the cell (Figure 1A). After obtaining the complete genomes of eight strains, taxonomic classification was carried out using GTDB-Tk. Based on classification results, strains M342 and W028 were identified as *Sulfitobacter mediterraneus* and *Sulfitobacter pontiacus*, respectively, while the other six strains could not be affiliated to any known species (Table 1). Other basic genomic information of these strains has been provided in Table 1. The genome sizes of the eight *Sulfitobacter* strains ranged from 3,499,419 bp to 4,223,094 bp. All strains had plasmids and one chromosome (Table 1). GC content varied from 56.25% to 61.48%, and the number of tRNA- and rRNA-coding genes was also different among the strains (Table 1). The strains were observed to have 3380 to 4077 open reading frames (ORFs) for protein coding (Table 1). An analysis of 31 essential protein-coding genes revealed that all *Sulfitobacter* strains had only one copy of these genes. Subsequently, a phylogenetic tree was constructed after concatenating these 31 genes. In this tree, eight strains were generally located on four branches (Figure 1B). However, a pairwise analysis of average nucleotide identity (ANI) revealed that the inter-strain distance ranged from 77.65 to 96.39, and only W002 and W074 had ANI > 95% (Figure 1C). The eight strains were classified into seven distinct species, as follows: M342 represented *S. mediterraneus*, W028 represented *S. pontiacus*, W002 and W074 represented a new species, while W027, W028, M368, S190, and S223 represented five distinct new species.

### 2.2. Metabolic Pathways

**Figure 2:**
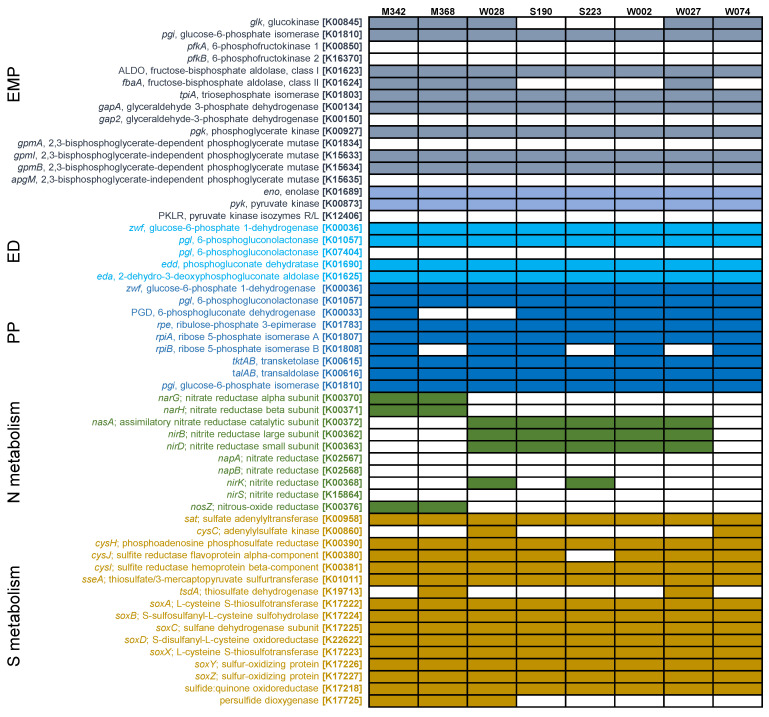
Distribution of central metabolism genes in *Sulfitobacter* genomes. Heatmap shows the genes related to the Embden–Meyerhof–Parnas (EMP), Entner–Doudoroff (ED), pentose phosphate (PP), nitrogen, and sulfur metabolism pathways. Blank boxes and colored boxes indicate the absence and presence of genes, respectively.

Based on the Kyoto Encyclopedia of Genes and Genomes (KEGG) annotations (Tables S1–S8), the metabolic pathways of the strains were analyzed and reconstructed. All fundamental genes related to the enzymes involved in the tricarboxylic acid (TCA) cycle were detected (Figure 2). Genes encoding key enzymes of the Entner–Doudoroff (ED) pathway were also identified, such as the genes related to glucose-6-phosphate 1-dehydrogenase (*zwf*, K00036), 6-phosphogluconolactonase (*pgl*, K01057), and phosphogluconate dehydratase (*edd,* K01690) (Figure 2). Similarly, genes encoding key enzymes of the pentose phosphate (PP) pathway were present in most of the genomes, such as the genes related to ribose 5-phosphate isomerase A (*rpiA*, K01807), ribose 5-phosphate isomerase B (*rpiB*, K01808), and glucose-6-phosphate isomerase (*pgi*, K01810) (Figure 2). However, the gene set required for the Embden–Meyerhof–Parnas (EMP) pathway was rather incomplete in all strains, lacking key genes encoding 6-phosphofructokinase 1 (*pfkA*, K00850) and 6-phosphofructokinase 2 (*pfkB*, K00850) (Figure 2).

For nitrogen metabolism, genes corresponding to the nitrate reductase alpha subunit (*narG*, K00370) and the beta subunit (*narH*, K00371) were identified in the M342 and M368 strains (Figure 2), along with the gene encoding nitrous-oxide reductase (nosZ, K00376) (Figure 2). Genes encoding nitrite reductase (*nirB*, K00362) and (*nirD*, K00363) were detected in S190, S223, W002, W027, and W028 (Figure 2). Interestingly, an assimilatory nitrate reductase encoding gene (*nasA*, K00372) was detected in the same five strains possessing *nirB* and *nirD*. These results indicated the various nitrate reduction processes in the isolated strains of *Sulfitobacter*, with or without energy production.

For sulfur metabolism, genes encoding a complete SOX system were detected in all strains, including the genes related to L-cysteine S-thiosulfotransferases (*soxA*, K17222; *soxX*, K17223), S-sulfosulfanyl-L-cysteine sulfohydrolase (*soxB*, K17224), the sulfane dehydrogenase subunit (*soxC*, K17225), S-disulfanyl-L-cysteine oxidoreductase (*soxD*, K22622), and sulfur-oxidizing proteins (*soxY*, K17226; *soxZ*, K17227) (Figure 2). In addition, sulfide:quinone oxidoreductase (K17218), which is responsible for sulfide oxidation, was detected in all genomes (Figure 2). These results suggested that the oxidation of the reduced form of the sulfur element might be an important energy production pathway in *Sulfitobacter* strains.

**Figure 3:**
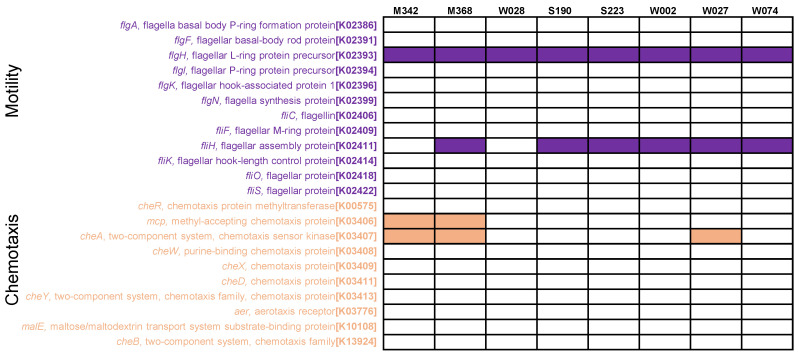
Distribution of motility and chemotaxis genes in *Sulfitobacter* genomes. Heatmap shows the genes related to motility and chemotaxis pathways. Blank boxes and colored boxes indicate the absence and presence of genes, respectively.

Given that *Sulfitobacter* strains are known for their ‘stick or swim’ switch, where they are either adapted to a non-motile biofilm or are migratory [16,17], we analyzed the pathways of motility and chemotaxis. According to the KEGG annotation, the motility and chemotaxis pathways were found to be rather incomplete in all the analyzed genomes (Figure 3). In terms of motility, only one gene (flagellar L-ring protein precursor, *flgH*, K02393) was present in all the genomes, while certain key genes of a functional flagella (e.g., flagellin; K02406; and flagella synthesis protein-coding gene *flgN*, K2399) were missing in all the genomes (Figure 3). Similarly, most of the genes for chemotaxis were not detected (Figure 3).

**Figure 4:**
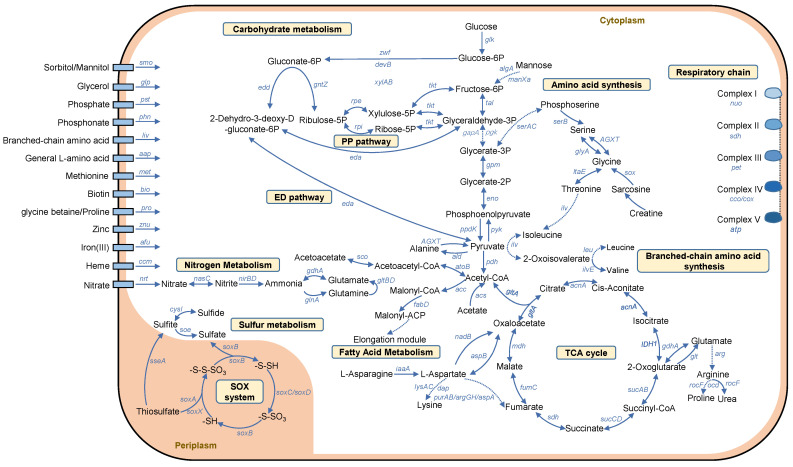
Reconstruction of key metabolic pathways in the representative strain W027. Dashed lines indicate simplified pathways.

Furthermore, the overall metabolic pathways were reconstructed and the profile of a representative strain (W027) is shown in Figure 4. Depending on the presence/absence of the key genes involved in central carbon metabolism, nitrogen, and sulfur metabolism, glucose was predicted to be metabolized through the ED and PP pathways, resulting in the generation of acetate and acetoacetate (Figure 4). Acetyl-CoA was predicted to either enter the TCA cycle or be metabolized to Malonyl-CoA and enter the fatty acid metabolism pathway (Figure 4). Furthermore, W027 was predicted to have the capability to oxidize sulfide, sulfite, and thiosulfate to sulfate in the cell periplasm (Figure 4). After uptake by the Nrt transporter, nitrate was indicated to be reduced to nitrite and then to ammonia (Figure 4). In addition, a complete respiratory chain was observed, which might allow electron transfer from complex I (NADH: ubiquinone oxidoreductase) to complex V (complexes and ATP synthase) following a steady gradient (Figure 4). In addition, several pathways of amino acid metabolism were reconstructed. For example, branched-chain amino acids (leucine, isoleucine, and valine) were suggested to be transformed to pyruvate, while lysine was suggested to be transformed to asparagine, aspartate, and then oxaloacetate (Figure 4).

### 2.3. Pangenome Analyses

**Figure 5:**
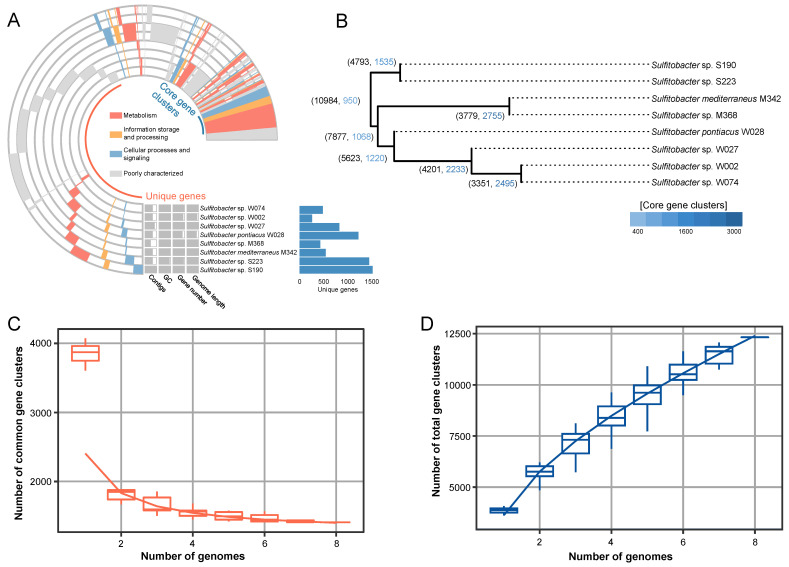
Pangenome analysis of *Sulfitobacter* strains. (**A**) Pangenome profile showing the functional distribution of core gene clusters and unique genes. (**B**) A single nucleotide polymorphism (SNP)-based tree showing the relationships between strains. (**C**) Accumulative curve of the number of core gene clusters and the number of genomes. (**D**) Accumulative curve of the total number of gene clusters and the number of genomes.

Pangenome analysis was conducted to identify the common and specific functions of the eight strains isolated in this study. According to the results of the integrated prokaryotes genome and pangenome analysis (IPGA), a total of 10,984 orthologous genes were identified after genome pooling. Clustering analysis revealed the presence of a large number of genome-specific genes, ranging from 259 (for W002) to 1573 (for S190). The core gene clusters mainly included the genes related to enzymes involved in metabolism, information storage and processing, cellular processes, and signaling, and were poorly characterized (Figure 5A). A single nucleotide polymorphism (SNP)-based tree was further built to visualize the relationship between the strains (Figure 5B). Consistent with the phylogenetic tree based on essential genes (Figure 1B), the SNP-based tree showed W002 and W074 as close relatives, while longer distances were found between other strains (Figure 5B). Additionally, the eight strains only shared 950 (8.6%) core gene clusters out of 10,984 gene clusters (Figure 5B). In particular, although S190 and S223 belonged to the same species, they only shared 1535 (32%) core gene clusters (Figure 5B), highlighting the significant genetic diversity within the genus *Sulfitobacter*. In addition, cumulative curves were drawn based on the number of common gene clusters (Figure 5C) and total gene clusters (Figure 5D). In terms of the number of common gene clusters, all genome pairs had less than 1900 common clusters, and therefore, the curve underwent a sharp decrease from genome number one to two (Figure 5C). In terms of the total number of gene clusters, the curve kept on rising from genome number one to eight, suggesting the addition of genome-specific genes (Figure 5D).

### 2.4. Potential of Strain for Biosynthesis of Secondary Metabolites

**Figure 6:**
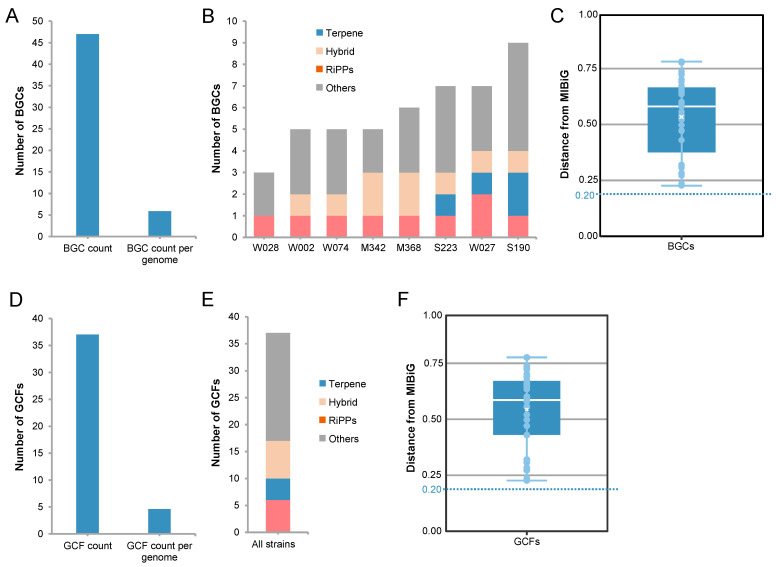
Summary and distribution of biosynthetic gene clusters (BGCs) in *Sulfitobacter* strains. (**A**) Total number and average number of BGCs in eight genomes. (**B**) Distribution of four types of BGCs in eight strains. (**C**) The minimum distance between BGCs in *Sulfitobacter* genomes and those in the Minimum Information about a Biosynthetic Gene cluster (MIBiG) database. White horizontal lines and crosses represent the median and mean, respectively. (**D**) Number of gene cluster *families* (*GCFs*) in the genomes. (**E**) Distribution of GCF categories in the genomes of eight strains. (**F**) The minimum distance between GCFs in *Sulfitobacter* genomes and those in the MIBiG database. White horizontal lines and crosses represent the median and mean, respectively.

The biosynthesis of secondary metabolites represents an important direction for bioresource mining. Therefore, the BGCs of the eight *Sulfitobacter* strains were analyzed by using antibiotics and secondary metabolite shell (AntiSMASH). A total of 47 BGCs were identified, with an average of 6 BGCs per genome (Figure 6A). These BGCs were generally distributed in four forms, as follows: terpenes, ribosomally synthesized and post-translationally modified peptides (RiPPs), hybrid BGCs, and others (Figure 6B). The strain S190 was observed to have the highest number (N = 9) of BGCs, including one RiPP, two terpenes BGCs, one hybrid BGC, and five other BGCs (Figure 6B). The novelty of these BGCs was evaluated by comparing them with the BGCs documented in the Minimum Information about a Biosynthetic Gene cluster (MIBiG) database. All BGCs showed minimum cosine distances of > 0.2, with a median value of 0.581 (Figure 6C), implying quite a low similarity with known BGCs.

The BGCs were further classified into gene cluster *families* (*GCFs*) based on a similarity index of 0.2. In total, 37 GCFs were identified from the 8 genomes, with an average of 5 GCFs per genome (Figure 6D). These GCFs included 6 RiPPs, 4 terpenes, 7 hybrid clusters, and 20 other clusters (Figure 6E). The similarity of GCFs with those in the MIBiG database was also determined by assessing the minimum distance between the BGCs in a given GCF and those in the MIBiG database. A median value of 0.585 was observed (Figure 6F), suggesting a low similarity of GCFs with known gene clusters.

**Figure 7:**
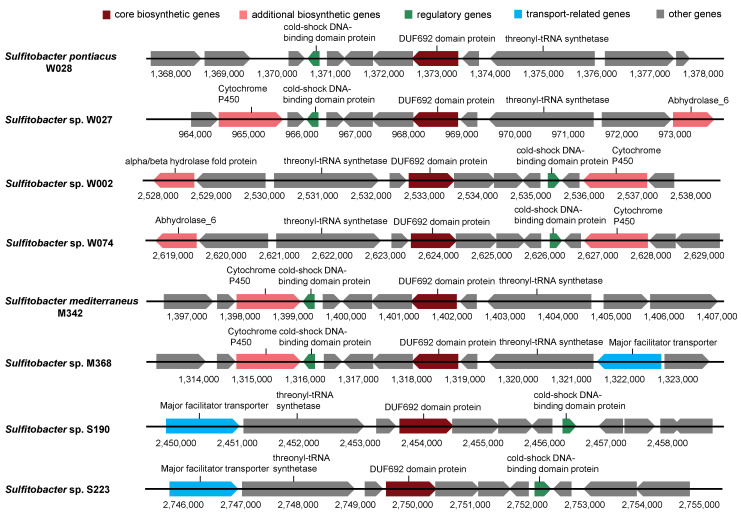
Arrangement of different types of genes within the BGCs of post-translationally modified peptides (RiPPs). Different gene categories are indicated by different colors.

Subsequently, the eight RiPP BGCs detected in the genomes were further investigated in detail. The length of the RiPPs was about 10,000 bp (Figure 7). Core biosynthetic genes and regulatory genes were found in all genomes, while additional biosynthetic genes were found in five genomes. The transport gene, the major facilitator transporter, was exclusively found in the genomes of M368, S190, and S223 (Figure 7). Additionally, all BGCs contained 8 to 9 other functional genes. Most of these genes varied in terms of length among the strains (Figure 7).

### 2.5. In Situ Biosynthetic Gene Expression

**Figure 8:**
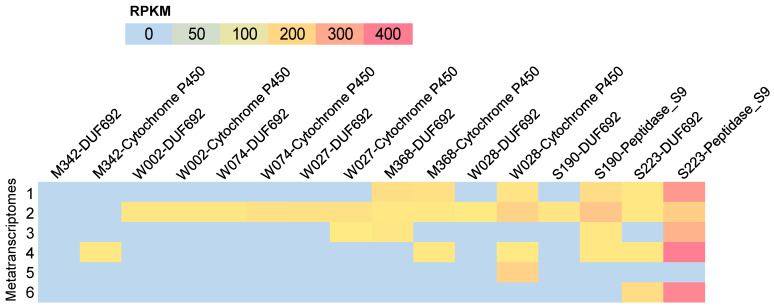
Expression levels of core genes related to RiPP synthesis in six metatranscriptomes derived from six biofilms. RKPM values, represented by a range of colors, indicate the reads per kilobase per million mapped.

RiPP production in strains was investigated under laboratory conditions using HPLC-MS/MS, after the overnight culturing of the strains in marine broth. However, no molecules with a molecular weight >10 kDa were detected. This implied that the RiPPs could not be produced by strains under a laboratory setting. Then, metatranscriptomic analysis was employed to explore the in situ expression of the core genes encoding key enzymes involved in RiPP synthesis in the strains. These genes were related to DUF692 enzymes in M368, W002, W074, W027, W028, S190, and S223; cytochrome P450 enzymes in M342, M368, W002, W074, W027, and W028; and S9 peptidases in S190 and S223 (Figure 8). The six metatranscriptomes used for this analysis were taken from our previous study [13], which corresponded to six biofilms on subtidal stones collected in September 2020, November 2020, January 2021, March 2021, May 2021, and July 2021. The basic information of these six metatranscriptomes is given in Table 2. Except for the strain M342, the genes encoding the DUF692 enzymes in all other strains were found to be expressed in at least one biofilm community (Figure 8). The genes related to the cytochrome P450 enzymes and the S9 peptidases of all strains were found to be expressed in at least one biofilm community (Figure 8). Interestingly, many of the investigated genes were observed to be expressed in the biofilm collected in Nov 2020. Among the studied genes, the S9 peptidase gene in S223 seemed to be the most active gene, as its reads per kilobase per million mapped reads (RPKM) values were over 200 in five biofilms (Figure 8). These findings revealed the in situ expression of RiPP BGCs in marine biofilms.

## 3. Discussion

In the present study, genomic analyses of novel *Sulfitobacter* strains have shed new light on their adaptability to a marine biofilm niche. The absence of a complete EMP pathway and the presence of the ED and PP pathways may be an adaptive strategy of these strains to environmental changes. This phenomenon is consistent with the characteristics of a recently discovered *Leisingera* strain (M597), which also belong to marine biofilm-derived roseobacters [18]. In the free-living state, the expression of key genes related to the EMP pathway significantly increased, along with the increase in temperature from 25 °C to 31 °C, whereas the expression of these genes was not affected in the biofilm state [18]. The augmentation of the EMP pathway was reported to be accompanied by the faster production of reactive oxygen species, causing damage to bacterial cells [19]. Consistently, the higher free energy enthalpy changes and the increased number of enzymatic reaction steps are thought to be limiting factors for the widespread distribution of the EMP pathway in marine bacteria [20,21]. In contrast, the ED pathway is more efficient in generating NADPH, which can enhance the cellular reducing power to antagonize the oxidative stress, and thus is essential to maintain a cellular homeostasis during environmental change [22]. The PP pathway provides reducing molecules and precursors for metabolic intermediates and amino acid biosynthesis, and it is very flexible and can coordinate these functions to adapt to constantly changing environments [23]. Therefore, the preference of ED and PP pathways, which may be a scenario of selective evolution of sugar metabolism, has been confirmed in many marine bacteria [24,25,26].

The diverse pathways of amino acid metabolism in the *Sulfitobacter* strains further support the adaptation to environmental change. This notion is in line with our recent study conducted on an unclassified *Roseobacteraceae* strain (M382) [27]. M382 employs amino acid metabolism for copiotrophic growth in carbon-rich environments, and this process is associated with the production of a higher biofilm biomass [27]. The presence of thiosulfate oxidation and dissimilatory nitrate reduction pathways in *Sulfitobacter* genomes suggests that these bacteria are versatile in energy production. As pointed out in our previous study [15], thiosulfate is likely to be consumed by marine biofilm bacteria as an important energy source. In addition, the genomic analysis of motility and chemotaxis pathways suggest that the marine biofilm *Sulfitobacter* strains are likely to be immobile, further indicating that they have adapted to the biofilm-associated lifestyle.

Furthermore, the findings of this study highlight the potential of *Sulfitobacter* strains in synthesizing secondary metabolites. These BGCs are clustered into several GCFs, which show high novelty when compared with the MiBIG database. These results were generally in line with previous studies evaluating the biosynthetic potential of marine microbiomes. Approximately 40,000 putative (mostly new) BGCs were revealed by analyzing around 10,000 genomes and more than 25,000 MAGs of marine microorganisms [28]. Hundreds of core peptides including novel antimicrobial peptides were identified from 11,572 BGCs [29]. Here, our results reveal the potential of marine biofilm *Sulfitobacter* strains in producing RiPPs. RiPP BGCs are often composed of genes encoding precursor peptides, modifying enzymes, main peptidases, and transporters [30,31]. RiPPs contain peptides with promising utilization, such as lanthipeptides, lasso peptides, graspetides, glycocins, linear azol(in)e-containing peptides, thioamitides, and streptamidine, and novel amidine-containing RiPPs [32]. Some of them display antimicrobial activity against a variety of human pathogenic bacteria [31,33,34]. For example, darobactin is a novel RiPP derived from nematophilic bacteria, and has a potent antibacterial activity against various Gram-negative bacteria [33]. However, RiPPs in *Sulfitobacter* strains were not well recognized in previous studies. The identification of BGCs in this study thus provides new implications for the discovery of novel RiPPs and antimicrobials.

Although RiPP production was not detected in *Sulfitobacter* strains under a laboratory setting, the transcription of key enzymes could be observed through a metatranscriptomic analysis of the biofilm. In particular, DUF692 enzymes represent an important family of post-translational modification enzymes involved in RiPP biosynthesis. These enzymes can mediate the chemical transformations that result in macrocycles and heterocycles [35]. Furthermore, P450 enzymes have diverse catalytic capabilities and are known to catalyze aromatic crosslinking by forming C–C, C–N, and C–O bonds [36]. The expression of the genes related to these enzymes in *Sulfitobacter* strains suggests that RiPPs may be utilized by *Sulfitobacter* strains to compete with other bacteria within marine biofilms, considering that many RiPPs can show a selective antimicrobial activity [37,38]. On the other hand, the expression of these genes implies the possibility of obtaining RiPPs through additional molecular approaches, such as BGC cloning and heterologous expression. However, considering that the synthesis of RiPPs involves the synergistic effects of multiple enzymes, exploring the functions and interactions of these enzymes to obtain final products are still challenging, which may require substantial efforts.

In addition, combining the results of the taxonomic classification and pangenome analyses of *Sulfitobacter* strains suggests that microbial species and their functional diversity are far from fully discovered and studied. The high species diversity is largely due to the diverse substrates available in seawater that allows the “proliferation after colonization” of numbers of rare species living in marine environments [14]. In addition to maintaining basic life functions through core genes, genetic differences revealed through the pangenome analyses indicate that the strain-specific genes may confer certain selective advantages, such as adaptation to biofilm microenvironments and antibiotic resistance [39]. Moreover, this genetic diversity can enhance the evolutionary ability of populations and represents an evolutionary strategy to cope with environmental pressures (e.g., salinity, temperature, and pH), thereby maintaining community stability [40,41].

## 4. Experimental Procedures

### 4.1. Biofilm Sampling and Strain Isolation

The marine biofilms were sampled from the surface of stones immersed in the subtidal zone of Qingdao (120.145, 39.915), China, using sterile cotton tips. The collected samples were suspended in sterile seawater and were immediately transferred to the laboratory for bacterial isolation. After vortexing, bacterial cells were resuspended in marine broth 2216E medium (Difco, Franklin Lakes, NJ, USA) and were diluted into a series of gradients (10^0^, 10^−1^, 10^−2^, 10^−3^, 10^−4^, and 10^−5^). The bacterial culture broth was inoculated onto 2216E agar plates and incubated at 25 °C until visible colonies appeared. Single colonies were picked up and examined using PCR and Sanger sequencing of 16S rRNA genes. A taxonomic classification of the strains was preliminarily conducted through a BLASTn search of 16S rRNA gene sequences against the Nucleotide database of the National Center for Biotechnology Information (NCBI), USA.

### 4.2. Observation of Bacterial Morphology with TEM

The cell morphology of the strains was observed using a TEM system (TESCAN VEGA 3, Tescan, Shanghai, China) at Qingdao University. For sample preparation, bacterial culture (grown for 16 h) was centrifuged for 5 min at 5000 rpm and bacterial cells were precipitated. These bacterial cells were washed three times with 0.1 M PBS (5000 rpm, 5 min). Subsequently, the cells were fixed with 2.5% glutaraldehyde for 2 h at room temperature, and then overnight at 4 °C. The fixed cells were further fixed for 1 h with 1% OsO₄ after washing with 0.1 M PBS. The fixed bacterial cells were dehydrated and embedded for slicing and dyeing. Finally, the dyed and dried bacterial cells were observed under a TEM.

### 4.3. Genomic Sequencing and Assembly

Whole genomic DNA was extracted from overnight-cultured strains using TIANamp Genomic DNA Kit (Tiangen, Beijing, China). Combining the long-read capability of PacBio with the high accuracy of Illumina, the extracted genome of each strain was subjected to PacBio and Illumina sequencing at the Novogene Bioinformatics Institute (China), generating 1 Gb and 2 Gb of clean data, respectively. The PacBio reads were filtered out for low-quality sequences with a length of less than 500 bp from the original data and were assembled using SMRT Link v5.0.1 [42] to obtain a preliminary assembly of the genome. Then, the genomes were corrected with the Illumina reads using Minimap2 [43]. Based on the coverage and annular length of the reads, chromosomal sequences were distinguished from plasmids. The completeness and the contamination of genomes were checked using CheckM (version 1.1.2) [44].

### 4.4. Basic Genomic Analyses

All genomic analyses were performed on a local server installed on the Linux Centos 7 platform. Taxonomic affiliation was carried out using the GTDB-Tk (version 0.3.2) database based on 120 single-copy marker genes [45]. The GC content of the genomes was calculated using Quast (version 5.0.2) [46]. The tRNA- and rRNA-coding genes were predicted using Prokka (version 1.14.6) [47]. Prodigal (version 2.60) was employed for the prediction of ORFs [48] and only close-end ORFs were returned. FastANI (version 1.31) was used for pairwise ANI comparison using default parameters [49].

### 4.5. Phylogenetic Tree Building

The 31 housekeeping genes (dnaG, frr, infC, nusA, pgk, pyrG, rplA, rplB, rplC, rplD, rplE, rplF, rplK, rplL, rplM, rplN, rplP, rplS, rplT, rpmA, rpoB, rpsB, rpsC, rpsE, rpsI, rpsJ, rpsK, rpsM, rpsS, smpB, and tsf) were extracted from the genomes of eight strains using AMPHORA2 [50]. Subsequently, the corresponding protein sequences of each gene were aligned using the *ClustalW* method in MEGA (version 7.0.26) [51]. The resulting 31 alignments were concatenated and subjected to phylogenic analysis in MEGA. The phylogenetic tree was built using the maximum likelihood method based on the Jones–Taylor–Thornton matrix-based model with 500 bootstrap replicates.

### 4.6. Metabolic Pathway Reconstruction and Pangenome Analysis

The protein sequences corresponding to all the ORFs derived from eight genomes were searched against the KEGG database (2022 version) purchased from Kanehisa Laboratory (Japan) using BLASTp (E-value < 1 × 10^−7^). Then, KEGG Mapper (https://www.genome.jp/kegg/mapper/) (accessed on 1 June 2022) was used to visualize the KEGG pathways. The presence of key functions was also examined manually. Subsequently, pangenome analysis was performed on IPGA [52] (https://nmdc.cn/ipga/) (accessed on 25 December 2021) to analyze the core genes (common genes in all strains), accessory genes (genes existing in some strains), and specific genes (genes exclusive for one strain).

### 4.7. Analysis of Biosynthetic Potential of Strains

BGCs of various secondary metabolites were predicted using antiSMASH (version 6.1.1) on the Linux platform [53]. Subsequently, BGCs were clustered into GCFs using BiG-SLICE (version 1.1.0) [54], based on cosine distance (cutoff = 0.2). The novelty of a given GCF was indicated by the minimum distance between the BGCs within the GCF and the BGCs documented in the MIBiG (version 3.1) database [55]. To describe the results, box plots were plotted using R (version 4.3.2).

### 4.8. Metatranscriptomic Analysis

Six metatranscriptomes were derived from six biofilms collected from the stones immersed in the subtidal zone in Qingdao in September 2020, November 2020, January 2021, March 2021, May 2021, and July 2021, respectively. The sample collection procedure was the same as mentioned in Section 4.1, except that the samples were immediately frozen in liquid nitrogen after transfer to the laboratory. RNA was extracted from each biofilm using the classical TRIzol method, followed by rRNA removal using biotin-labeled oligonucleotides. RNA-Seq libraries were prepared with NEBNext Ultra RNA Library Prep Kit (New England Biolabs, Ipswich, Massachusetts, USA). Subsequently, RNA sequencing was performed on Novaseq 6000 at Novogene Bioinformatics Institute (Beijing, China) with the PE150 strategy, resulting in more than 60 Gb data per sample with 150 bp paired-end reads. The NGS QC Toolkit (version 2.0) [56] was used to check the quality of the sequence and to remove low-quality (quality score < 20) reads. Gene expression levels were determined by indexing the ORF sequences of targeted genes using bowtie-build in Bowtie (version 2.5.2) [57]. The metatranscriptomic reads were mapped onto ORFs and the mapped reads were analyzed using SAMtools (version 1.11) [58]. Finally, the RPKM value was calculated for each core gene using the following formula:

RPKM = number of recruited reads/(gene length/1000 × total number of reads/1,000,000).

### 4.9. Data Availability

The complete genomes of eight *Sulfitobacter* strains (M368, W027, W002, M342, W028, S190, S223, and W074) were deposited in NCBI with accession numbers CP081113-CP081115, CP083564-CP083568, CP081126-CP081129, CP081109-CP081112, CP081116-CP081119, CP081120-CP081125, CP083560-CP083563, and CP081130-CP081133, respectively.

## 5. Conclusions

The complete genomes of eight *Sulfitobacter* strains isolated from marine biofilms were analyzed in this study. The genomes were observed to have the complete gene sets for the ED and PP pathways. Genes responsible for amino acid metabolism were detected in the strains, suggesting the presence of an optimized central carbon metabolism, which is important for bacterial adaptation to environmental changes. The presence of genes related to thiosulfate oxidation and nitrate reduction indicated accessory energy production approaches in strains. Moreover, BGCs, including those encoding RiPPs, were detected in the *Sulfitobacter* genomes. From a comparative genomics perspective, this study offers new insights into the metabolic characteristics of *Sulfitobacter* strains associated with marine biofilms, contributing valuable knowledge to microbial adaptation, as well as the biochemical cycling of carbon, nitrogen, and sulfur. Based on the perspective of metabolites, the identification of undescribed BGCs from novel species constitutes a precious resource library for the development of novel marine pharmaceuticals. In the future, it is imperative to continue enhancing the marine microbial BGC database by integrating genomic information, functional annotations, and metabolomics. Additionally, efforts should be directed towards improving the yield of BGC products through techniques such as heterogeneous expression and gene editing.

## Figures and Tables

**Table 1:** Genomic information of eight strains.

Strains	Contigs	Chromosomes	Plasmids	Total Length (bp)	GC Content (%)	CDS	rRNA	tRNA	ORFs	KEGG-Annotated ORFs	NCBI Accession Number
M368	3	1	2	4,014,743	58.52	3944	6	44	3947	2942	CP081113-CP081115
W027	5	1	4	4,147,109	60.24	4013	9	52	4022	3001	CP083564-CP083568
W002	4	1	3	3,707,314	61.42	3605	12	51	3611	2691	CP081126-CP081129
M342	4	1	3	4,223,094	58.01	4073	6	46	4077	3046	CP081109- CP081112
W028	4	1	3	3,499,419	60.45	3375	9	49	3380	2553	CP081116-CP081119
S190	6	1	5	3,966,771	61.48	3867	6	47	3872	2836	CP081120-CP081125
S223	4	1	3	4,030,883	56.25	3800	6	44	3802	2773	CP083560-CP083563
W074	4	1	3	3,976,848	60.96	3880	12	52	3888	2872	CP081130-CP081133

**Table 2:** Data information of six biofilm metatranscriptomes.

Sample Name	Sampling Time	NCBI Accession Number	Clean Reads	Read Length (bp)	Contigs	Average Length of Contigs (bp)	ORFs
Metatranscriptome-1	September 2020	SAMN21619182	220,546,791 × 2	150	2,515,854	510.4	2,999,230
Metatranscriptome-2	November 2020	SAMN21619183	230,543,188 × 2	150	3,111,162	498.8	3,703,787
Metatranscriptome-3	January 2021	SAMN21619184	241,275,710 × 2	150	3,316,686	476	1,242,031
Metatranscriptome-4	March 2021	SAMN21619185	208,807,982 × 2	150	3,845,996	513.2	1,537,969
Metatranscriptome-5	May 2021	PRJNA753157	218,063,438 × 2	150	855,508	626.1	402,727
Metatranscriptome-6	July 2021	PRJNA753157	272,768,371 × 2	150	1,124,063	310.9	1,189,877

## Data Availability

The authors declare that all relevant data supporting the findings of this study are available within the article and its Supplementary Material Files. The complete genomes of eight *Sulfitobacter* strains (M368, W027, W002, M342, W028, S190, S223, and W074) were deposited in the NCBI database with accession number CP081113-CP081115, CP083564-CP083568, CP081126-CP081129, CP081109-CP081112, CP081116-CP081119, CP081120-CP081125, CP083560-CP083563, and CP081130-CP081133, respectively.

## Supplementary Materials

The following supporting information can be downloaded at: https://www.mdpi.com/article/10.3390/md22070289/s1, Table S1: Gene functional annotation of M342; Table S2: Gene functional annotation of M368; Table S3: Gene functional annotation of S190; Table S4: Gene functional annotation of S223; Table S5: Gene functional annotation of W002; Table S6: Gene functional annotation of W027; Table S7: Gene functional annotation of W028; Table S8: Gene functional annotation of W074.
